# Hepatocellular carcinoma surveillance in Europe: challenges and lessons from global practice

**DOI:** 10.1016/j.lanepe.2026.101852

**Published:** 2026-09-03

**Authors:** Maria Qurashi, Maria Buti, Grace Lai-Hung Wong, Huma Qureshi, Masatoshi Kudo, Tawesak Tanwandee, Chien-Jen Chen, Ashraf Omar Abdelaziz, Mohamed AbdAllah, Jessica Howell, Vanessa Hebditch, Amit G. Singal, Peter Jepsen, Rohini Sharma

**Affiliations:** a Department of Surgery and Cancer, Imperial College London, United Kingdom; b Liver Unit Hospital Universitario Valle Hebron, Barcelona, Spain; c Medical Data Analytics Centre, Department of Medicine and Therapeutics, The Chinese University of Hong Kong, Hong Kong SAR, China; d Doctor's Plaza, Clifton, Karachi, Pakistan; e Department of Gastroenterology and Hepatology, Kindai University Faculty of Medicine, Osaka, Japan; f Division of Gastroenterology, Department of Medicine, Faculty of Medicine, Siriraj Hospital, Mahidol University, Bangkok, Thailand; g Genomics Research Center, Academia Sinica, Taipei, Taiwan; h Department of Endemic Medicine and Hepatogastroenterology, Cairo University, Cairo, Egypt; i Medical Research Division, National Research Centre, Cairo, Egypt; j Burnet Institute, Melbourne, Australia; k Department of Gastroenterology, St Vincent’s Hospital Melbourne, and Department of Medicine, University of Melbourne, Australia; l British Liver Trust, United Kingdom; m Department of Internal Medicine, University of Texas Southwestern Medical Center, Dallas, TX, USA; n Department of Hepatology and Gastroenterology, Aarhus University Hospital, Aarhus, Denmark; o Department of Clinical Epidemiology, Aarhus University Hospital, Aarhus, Denmark; p School of Medicine, University of Nottingham, Nottingham, England, United Kingdom

**Keywords:** Hepatocellular carcinoma, Surveillance, Uptake, Europe, Public health

## Abstract

Hepatocellular carcinoma (HCC) is a leading cause of cancer-related mortality worldwide and a public health concern, with 75% of cases considered preventable. Biannual liver ultrasound, with or without serum alpha-fetoprotein, is recommended as surveillance for patients at high risk of HCC. While the rationale for surveillance is strong and it continues to be recommended by all professional societies, there is ongoing debate about the strength and relevance of the evidence supporting surveillance for all populations with cirrhosis. European surveillance uptake is suboptimal, with high rates of late-stage diagnosis and subsequent poor prognosis. In this paper, we provide an overview of surveillance focusing on Europe and draw lessons from across the world. We discuss lessons learnt from the implementation of national and coordinated surveillance programmes in Asian countries and the global challenges resulting from the changing epidemiology of chronic liver disease, particularly the rise of MASLD. We consider the patient perspective, including stigma and inequalities in access and delivery. We explore emerging approaches to surveillance, including biomarkers, abbreviated MRI and risk-stratification, which may improve early diagnosis. Finally, we address the lack of funding and political willpower arising from an insufficient evidence base.

## Introduction

**Search strategy and selection criteria**

References for this paper were identified through searches of PubMed with the search terms “hepatocellular carcinoma”, “surveillance”, “uptake”, “incidence”, “diagnosis” from 2010 until 2026. Additional articles were identified through searches of the authors’ own files. The final reference list was generated on the basis of originality and relevance to the scope of this paper.

**Key messages**

• The evidence for mortality improvement with surveillance originates from a single randomised controlled trial in patients with hepatitis B. There have been no subsequent randomised studies. Meta-analysis illustrates that HCC surveillance in patients with liver cirrhosis increases early diagnosis, receipt of curative treatment and improves survival. There continues to be debate around the strength and generalisability of existing evidence supporting surveillance.
• Biannual surveillance uptake is low (<10% from a recent meta-analysis), particularly in Europe and the Americas, where there are no national surveillance programmes and ongoing debate about the utility of surveillance. Barriers to surveillance uptake can be patient-related, clinician-related and healthcare system related.
• Globally, most nations practice 6-monthly ultrasound and AFP with some adding biomarkers including PIVKA-II, GAAD and AFP-L3. There is wide variation in funding models, with many European nations offering free surveillance, whereas other countries utilise insurance models with differing coverage.
• The steep rise of MASLD and related complications represents a challenge: MASLD-related HCC can arise in the absence of cirrhosis and MASLD is projected to soon be the leading cause of HCC. This necessitates a more cost-effective model of surveillance.
• The future of HCC surveillance will likely be shaped by: (i)risk stratification; (ii)novel biomarkers; and (iii)alternative surveillance imaging, with abbreviated MRI being tested in several international surveillance trials.

HCC is the most common form of primary liver cancer and represents a growing public health concern. HCC is the sixth most common cancer worldwide,^1^ with 841,080 new cases in 2018, and the third leading cause of cancer related death, responsible for 746,000 deaths every year. Unlike other cancer types, incidence and mortality rates of HCC continue to rise.^2^

Ninety per cent of cases arise on a background of chronic liver disease (CLD). There are approximately 800,000 new cases diagnosed annually worldwide,^1^ of which three quarters are considered preventable. HCC prognosis remains poor, with five-year survival rates less than 20%^3^; this is unchanged in the last decade despite advances in therapeutics. Curative options rely on early diagnosis of cancer, but in Europe, less than 40% of cases are diagnosed early.^4^

Treatment follows the Barcelona Clinic Liver Cancer guidelines (Fig. 1).^5^ Patients diagnosed with early-stage disease can be offered treatments with curative intent^5^ such as tumour ablation, tumour resection or liver transplantation resulting in a median overall survival of >5 years. In comparison, the goal of treatment in intermediate and advanced stage disease is palliation.

**Fig. 1:**
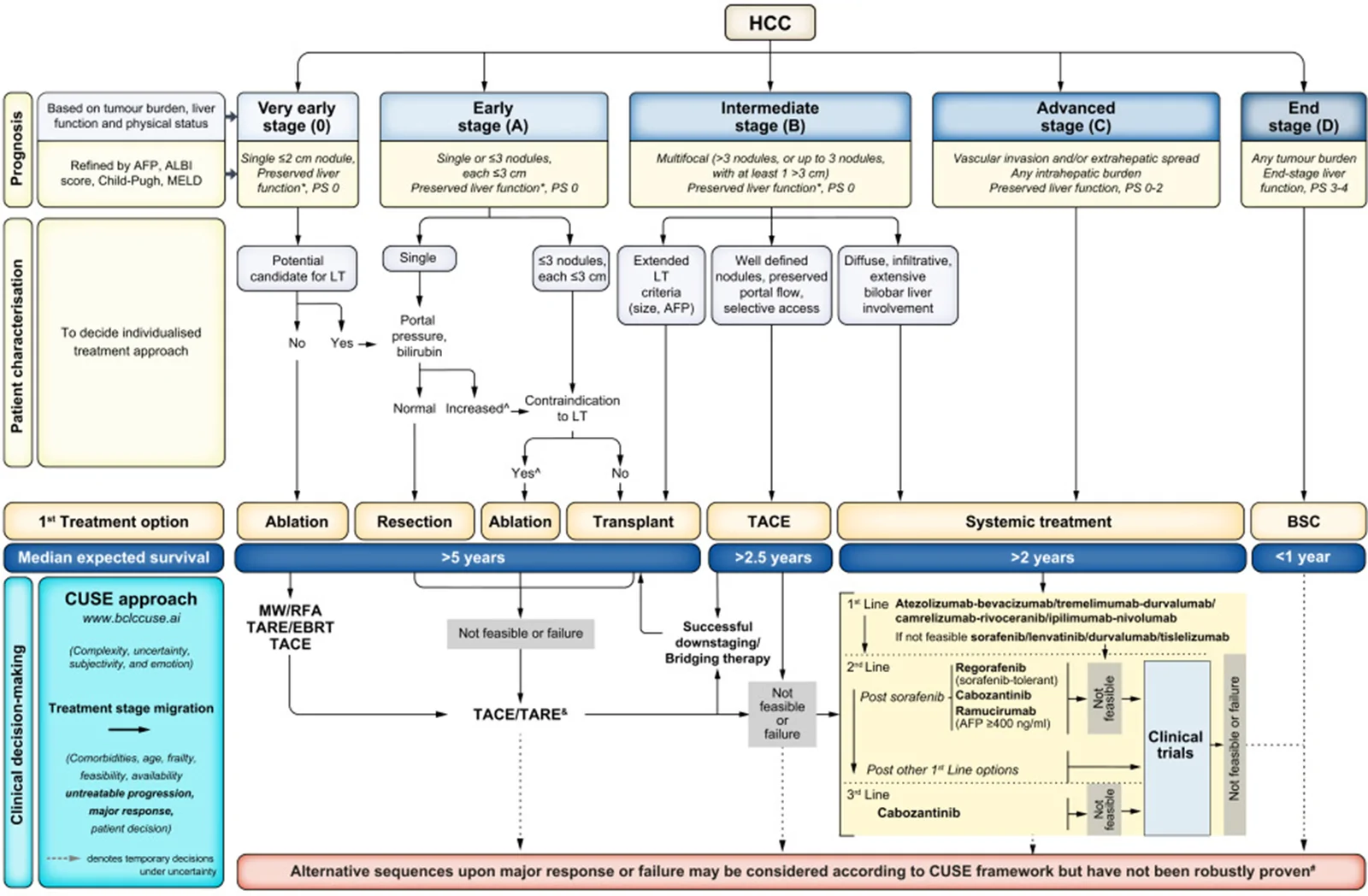
The updated 2026 Barcelona Clinic Liver Cancer treatment strategy for HCC.^5^

## Surveillance

**Panel 1**

Both screening and surveillance are secondary prevention methods forming the cornerstone of public health in cancer. *Screening* involves testing the apparently healthy, asymptomatic population for risk factors, or early signs of, disease; for example, screening all women over the age of 50 for breast cancer.

The principle of *surveillance* is to repeatedly test asymptomatic individuals who are at risk of developing a disease, with the aim of early detection; for example, annual colonoscopy for people with a family history of bowel cancer. The ideal surveillance test should be minimally invasive, affordable, acceptable to patients and have a high sensitivity and specificity.^6^ As HCC arises almost exclusively in patients with CLD, there is a well-defined target population who may benefit from the implementation of surveillance. HCC surveillance is recommended by all international professional bodies, with guidelines summarised in Table 1. The goal of surveillance is to reduce HCC-related mortality by offering curative treatments, which are available only when early-stage diagnosis is made.

**Table 1:** International professional society guidelines on HCC surveillance.

Professional society guidelines	Target surveillance population	Surveillance modality and frequency
EASL 2025^3^	- Cirrhosis (Child Pugh A and B) - Cirrhosis Child Pugh C, awaiting liver transplantation - Chronic intermediate or high risk^a^ HBV - Advanced fibrosis (Metavir > 3) of any aetiology	Liver ultrasound ± serum alpha-fetoprotein 6 monthly
AASLD 2023^7^	- Cirrhosis (Child Pugh A and B) - Cirrhosis Child Pugh C awaiting transplantation - High risk chronic HBV (black ethnicity age > 20, Asian males age > 40, Asian females age > 50, family history of HCC)	Liver ultrasound ± serum alpha-fetoprotein 6 monthly
APASL 2017^8^	- Cirrhosis (any Child Pugh stage) - High risk chronic HBV^a^ (Africans age > 20, Asian males age > 40, Asian females age > 50, family history of HCC)	Liver ultrasound + serum alpha-fetoprotein 6 monthly

EASL, European Association for Study of the Liver; AASLD, American Association for the Study of Liver Disease; APASL, Asian Pacific Association for the Study of the Liver; HBV, Hepatitis B virus.

a Risk as defined by the PAGE-B scoring system.^9^

The risk of developing HCC in CLD varies between 1 and 8% per annum, depending on the underlying pathology, for example, incidence rates per annum are typically 1% in alcohol-related liver disease (ArLD) cirrhosis, 2% in hepatitis B virus (HBV) and 3–8% in hepatitis C virus (HCV).^10^ There is however heterogeneity in risk between nations, which in turn affects national surveillance guidance and practices.^11^ Danish health registries have found an annual HCC incidence at 0.7% in those with ArLD cirrhosis leading to recommendations that this cohort do not require surveillance. Meanwhile, prospective cohort studies in Spain and France have found annual incidence rates between 2.1% and 3.2% in ArLD,^12^^,^^13^ thus supporting surveillance.

## Evidence for surveillance

The evidence base is drawn largely from a single randomised controlled trial in China,^14^ assessing HCC-related mortality in patients with hepatitis B (presumably without cirrhosis). The trial demonstrated that 6-monthly ultrasound of the liver ± serum alpha fetoprotein (AFP) measurement reduced HCC-related mortality by 37%. Populations with non-cirrhotic viral hepatitis, who have a higher risk of developing HCC but fewer comorbidities, are likely to benefit the most from surveillance. Further randomised controlled trials have been considered unfeasible or unethical,: a 2011 Australian study tested the feasibility of a randomised controlled trial which would randomise some patients to no surveillance, with 99.5% of patients declining randomisation.^15^ This feasibility study had several shortcomings, but the impact of its results has created a prevailing sentiment and as a result robust, prospective trial evidence in patients with cirrhosis is lacking.

Novel data on surveillance benefits in cirrhosis is largely drawn from cohort studies or modelling. A meta-analysis^16^ of 59 international studies involving 145,396 patients demonstrated that HCC surveillance was associated with early-stage diagnosis (RR 1.86), curative treatment receipt (RR 1.83) and overall survival (HR 0.67) among patients who developed HCC, when adjusted for lead-time bias. Consequently, biannual surveillance in those with cirrhosis is recommended by all international professional bodies (Table 2), with adherence to biannual surveillance being associated with early diagnosis and improved survival outcomes.^22^

**Table 2:** HCC surveillance practices by country.

Country	Modality of surveillance	Biomarkers used beyond AFP?	National surveillance programme?	Surveillance uptake	% of early stage HCC diagnosed^a^
United Kingdom	6-monthly ultrasound ± serum AFP	No	No	17%	20%
France^17^	6-monthly ultrasound ± serum AFP	No	No	8%	28%
Italy^18^	6-monthly ultrasound ± serum AFP	No	No	46%	34%
Spain	6-monthly ultrasound ± serum AFP	No	No	47%	55%
Netherlands^19^	6-monthly ultrasound ± serum AFP	No	No	27%	32%
Denmark	6-monthly ultrasound ± serum AFP, only in viral hepatitis	No	No	–	–
Norway^20^	6-monthly ultrasound + serum AFP	No	No	4%	15%
Sweden^21^	6-monthly ultrasound	No	No	33%	35%
USA	6-monthly ultrasound + serum AFP Some centres use CT or MRI	Occasional use of AFP-L3 and DCP, not in guidelines	No	<20%	
Japan	3–6 monthly ultrasound (depending on risk) + serum AFP	PIVKA-II AFP-L3	Yes	80%	65%
Hong Kong	6-monthly ultrasound + serum AFP	PIVKA-II GAAD in cirrhosis and MASLD	No	–	50%
Taiwan	6-monthly ultrasound + serum AFP	PIVKA-II	Yes	75%	66%
Thailand	6–12 monthly ultrasound + serum AFP	GAAD in some centres	No	Unknown, 5%	53%
Pakistan	6-monthly ultrasound + serum AFP	No	No	<10%	12.8%
Egypt	6-monthly ultrasound + serum AFP P	No	Yes	–	–
Australia	6-monthly ultrasound + serum AFP	PIVKA-II and GAAD approved, but not covered by Medicare	No	40–50%	47%

a National estimates for early stage HCC, defined as BCLC A or 0.

There are notable criticisms of the current evidence base. Concerns have been raised about whether the effect size seen in a group of largely non-cirrhotic hepatitis B carriers can be generalised to older populations with established cirrhosis, who may be less likely to tolerate and benefit from any treatment offered.^23^ A reduction in risk of HCC-related mortality may be of limited real-world benefit in patients with advanced liver disease facing multiple competing causes of death. This has been observed in Danish health registry studies examining causes of death in ArLD, where HCC was found to have only a minor impact on mortality.^24^ This is also a challenge in patients with MASLD, where cardiovascular disease is key contributor to mortality.

The modality of surveillance also draws criticism: ultrasound is operator-dependant and has poor sensitivity (47%, 95% CI: 33–61%) for early disease, which is reduced further in patients with increased adiposity or advanced cirrhosis.^3^ Twenty per cent of surveillance ultrasounds are considered inadequate to visualise the liver, a particular concern given the rising incidence of MASLD and obesity.^25^ In cases with poor ultrasound visibility, guidelines advise that CT or MRI could be utilised.^7^ However, there are no randomised controlled trial data on the performance of cross-sectional imaging as a surveillance tool, and it is recognised that CT and MRI are likely to be limited by expense, cumulative gadolinium and radiation risk.

Finally, the balance of harm and benefit from HCC surveillance has not been examined on a large scale. Harms may be classified into physical (unnecessary radiation exposure and biopsies in false positive cases), financial (cost to the patient of repeated imaging, including travel and time off work) and psychological distress in patients undergoing testing.^26^^,^^27^ One study from the United States found physical harms of surveillance occurred in 27% of patients,^26^ although other studies have reported lower rates,^28^ leading to concerns about the benefit:harm ratio in certain patient groups. The clinical significance of psychological and financial harms may be more clinically relevant and warrants further evaluation.^27^^,^^29^ Further, the net benefit of surveillance likely varies based on baseline risk of developing HCC.^30^ Clear defining and quantification of risk may empower clinicians and patients to make informed, personalised decisions about surveillance. Clearly, there is a need for a new randomised control trial of surveillance versus no-surveillance. However, it is unlikely that this will be performed; the numbers of patients required would be in the thousands, patients would be unlikely to accept randomisation to the no-surveillance arm and there would be contamination of the no-surveillance arm due to the pervasive use of surveillance in the community. Despite controversies, HCC surveillance continues to be recommended by all professional bodies for those with cirrhosis, and yet the uptake is low.

## Surveillance uptake and challenges

HCC surveillance is recognised to be a complex, multistep process with several points of failure.^31^ A recent meta-analysis of 49 studies including over 1.2 million patients assessed surveillance uptake in patients at risk of HCC across the world. The authors reported that utilisation of any surveillance in eligible patients was 54%, but less than 10% of eligible patients received biannual surveillance^32^ in line with guidance. Biannual surveillance receipt in patients with cirrhosis was lowest in the USA (5.8%) and highest in Europe (62%). Additionally, pooled analysis of over 40,000 patients diagnosed with HCC reported that only 36% had undergone previous ultrasound surveillance.

Barriers to surveillance uptake can be categorised into patient, clinician and healthcare-system related. Patient barriers include a lack of awareness of risk,^33^ logistical and financial constraints, in addition to sociodemographic determinants such as ethnicity and education level.^34^ Clinician-reported barriers were a lack of knowledge, limited time and scepticism of the surveillance guidelines, resulting in imaging not being organised.^35^^,^^36^ Barriers relating to healthcare systems include a lack of national surveillance programme and supporting infrastructure.^37^

Most work in this area has been observed in American cohorts, which are not reflective of European or more global challenges. The European model of state healthcare funding means that the cost of surveillance imaging is less likely to affect uptake. Sociodemographic determinants of surveillance have been examined in the UK, where patients of low socioeconomic status or under the care of a non-specialist hospital^38^ were less likely to receive biannual surveillance. This supports French registry data showing that low socioeconomic status is associated with failed surveillance, despite universal health coverage.^17^ Additionally, the mode of acquiring liver disease may affect decisions about surveillance funding and thus impact on surveillance uptake rates: in Asia, most viral hepatitis cases are acquired through vertical or nosocomial transmission prompting the implementation of universal vaccination and surveillance programmes.^39^ Conversely, in Europe and North America where there is a higher percentage of intravenous drug users with viral hepatitis and alcohol misuse as the cause of CLD, there may be less motivation to fund HCC surveillance programmes.^23^ Adherence in those with unstable housing is another challenge, possibly requiring alternate surveillance strategies which again may not be funded in a resource-scare system.^40^

Europe’s Beating Cancer Plan, established in 2021, was a political and financial commitment towards transforming cancer prevention and treatment.^41^ It earmarked €4 billion towards actions addressing cancer, with a special focus on promoting early detection. However, in reference to HCC, the European Commission states surveillance “*screening of people at elevated risk* [of HCC] *entails uncommon but serious harms, and might not result in decreased mortality*”.^41^ It supports primary prevention only, with policies to reduce alcohol consumption, strengthen vaccination programmes and improve access to healthier diets. A similar situation has arisen in the USA: the National Cancer Institute concludes, based on Chinese RCT, that surveillance “*does not result in a decrease in mortality from hepatocellular carcinoma*”. On review of the same evidence, the European Commission and National Cancer Institute have drawn conflicting conclusions from professional bodies on the perceived utility of HCC surveillance.

This creates a conflict whereby informed patients and advocacy groups lobby for surveillance whereby many clinicians remain skeptical awaiting formal guidance from national programmes. Inevitably, patients are caught in the middle of this debate. It leaves cirrhotic and viral hepatitis patients in a state of flux and receiving variable care; the impact of this cannot be understated, particularly as liver disease is a disease of inequality, affecting some of the most marginalised members of society.

Within this context of policy and evidence debate, we highlight several case studies globally to understand differing surveillance patterns. Within Europe we discuss surveillance in Spain, as leaders in the management of HCC; Denmark, who have made a conscious decision not to survey those with ArLD; and the UK, the largest universal healthcare model, as it moves towards the introduction of a national surveillance programme.

Japan is the gold standard in HCC surveillance; the first country to implement a national surveillance programme and with the highest rates of early detection in the world. This has resulted in over 90% adherence to surveillance, translating into a 5-year survival rate of 43%, more than double the survival seen in Europe.^42^ We compare to other Asian nations, including those without national surveillance programmes, and finally the USA as the biggest global spender on healthcare. Central to the debate is the patient experience, often ignored.

## The position of surveillance in Europe

### United Kingdom

The UK is amongst the worst outcomes of hepatobiliary cancers amongst wealthy nations,^43^ with less than 20% of patients with HCC receiving curative therapies. The most common route to HCC diagnosis is emergency presentation, which was associated with reduced 1-year mortality and lower likelihood of curative treatment.^44^ Late-stage diagnosis is associated with poor quality of life and more frequent hospital admissions, leading to significant economic impact.^45^ A recent study reported biannual surveillance uptake of 17%, with lower uptake in patients experiencing deprivation and under the care of non-specialist hospitals.^38^

There is no formal surveillance programme; surveillance is organised through specialist hepatology and gastroenterology centres, meaning that patients are often not invited to regular appointments. UK clinicians report complex barriers to providing HCC surveillance, including skepticism of guidelines and challenges in providing timely care within a healthcare system in “critical condition”.^35^^,^^46^

The scale of the problem is also unknown; there is no national cirrhosis registry.^37^ Recognising this issue, England has commenced a community pilot programme to identify those with CLD with subsequent referral for management and HCC surveillance if required. This programme has identified 7892 patients for referral to hepatology but as yet lacks follow-up data on HCC-related mortality. Importantly, no formal health economic data has been reported central to the programme’s sustainability.

### Denmark

Surveillance is offered to those with viral hepatitis or who are at high risk based on the PAGE-B score.^9^ Danish guidelines state that there is insufficient evidence to recommend surveillance of patients with cirrhosis from other causes. There is no national data on uptake of surveillance.

The major challenge in Denmark is the insistence on stronger evidence before it can be recommended to the larger groups of patients, i.e., those with alcohol-or MASLD-related cirrhosis. It is difficult to produce that evidence because of the expected low incidence of HCC among at-risk groups (0.7% per year among Danish outpatients with ArLD).^16^ Denmark is currently producing stronger evidence by introducing—in one region—surveillance to all patients with cirrhosis. Going forward, incidence and HCC-related mortality between the surveillance region and the other regions will be compared. It is expected that this study will provide an up-to-date estimate of HCC incidence in a Danish cirrhosis population offered surveillance, but statistically significant differences in HCC-related mortality are not anticipated due to the small population size.

### Spain

Surveillance is implemented through a risk-stratified, clinician-led model embedded within hepatology services (Table 2).^3^ Individuals with advanced fibrosis are increasingly considered for surveillance on a personalised basis, reflecting a shift toward risk-adapted strategies.^47^

Within specialised care, uptake approaches 80% but important gaps persist at the population level. National data indicate that only 47% of cases are detected through surveillance, largely due to underdiagnosis of CLD, suboptimal adherence, and missed referrals.^48^ Regional variability further contributes to inconsistent implementation.

There is variation in surveillance approaches across Europe (Table 2) which is of interest given that most nations are high-income and have comparable expenditure on healthcare. This suggests differences in both interpretation and application of the evidence based on local epidemiology of liver disease.

## Learnings from other nations

### Japan

A nationwide surveillance program was established during the 1980s targeting patients with chronic viral hepatitis and cirrhosis who are at high risk for developing HCC. The Japan Society of Hepatology (JSH) worked closely with the Japanese government and the Ministry of Health, Labour and Welfare to strengthen awareness and establish national surveillance strategies.^49^

According to the JSH Guidelines, patients classified as super-high-risk, including those with HBV or HCV related cirrhosis, are offered ultrasound and measurement of three tumour markers—AFP, AFP-L3, and PIVKA-II—every three to four months, with optional dynamic CT or Gd-EOB-MRI imaging once or twice annually. High-risk patients, such as those with chronic HBV or HCV, or non-viral cirrhosis, are recommended to receive surveillance every six months. Japan is unique in routinely using all three tumour markers in clinical practice.^50^

A key strength of the Japanese system is that ultrasound examinations are often performed directly by hepatologists and gastroenterologists rather than radiologists, streamlining the pathway under a single specialty. Combined with tumour marker monitoring and careful risk stratification, this approach has enabled earlier HCC diagnosis.

The JSH further strengthened national awareness by launching an HCC eradication campaign in 1999. Educational meetings are held annually for healthcare professionals and the general public across all prefectures to improve recognition of risk factors and emphasize the importance of surveillance. Additionally, Japan established a nationwide HBV and HCV testing system available at hospitals, clinics, and public health centers, while workplace and community health checkups routinely include viral hepatitis screening. Electronic medical record alert systems in major hospitals also encourage referral to hepatologists when hepatitis virus positivity is identified outside liver-related departments.

As a result of these coordinated efforts among government agencies, academic societies, and healthcare institutions, Japan has achieved one of the world’s most effective HCC surveillance systems, covering more than 80% of high-risk individuals where 68% of HCC cases are detected as solitary tumours, 53% are identified when lesions are 3 cm or smaller, and 65% are diagnosed at BCLC stage 0 or A.

In comparison, for example, Australia, which has comparable access to hepatitis B and C treatment but does not have a national surveillance registry, only 47% are diagnosed with early stage HCC, highlighting the utility of a national surveillance programme.^51^

### The rest of Asia and the Middle East

HCC control in Taiwan, like nations such as Egypt and Pakistan, has been focused on the elimination of HBV and HCV, with considerable success^52^: a national HBV immunisation programme^53^ led to a significant reduction in HCC rates.^54^ The national liver cancer surveillance programme, built upon the HBV and HCV screening programmes, reimburses for surveillance in those with chronic viral hepatitis (Table 2). However, one-third of patients are still diagnosed at an advanced stage. The rise of MASLD-HCC is an increasing concern, particularly in resource poor nations. As stated before, there is little evidence supporting surveillance in this population, particularly with the risk of competing mortalities. Taiwan has developed a specific strategy to address rising MASLD cases, launching the national 888 programme on the clinical management of hypertension, hyperglycemia and hyperlipidemia in 2024. The programme aims to reach three targets of 80% by 2032: enrolling patients diagnosed with the conditions into a formal medical care and tracking network; ensuring those in the network receive personalized lifestyle counselling; effective control for these conditions among participants. Achieving these aims may impact upon both MASLD progression and HCC incidence.

Another approach is the use of risk-stratification. In Thailand, the application of risk scores, particularly in non-cirrhotic HBV patients may reduce the surveillance-eligible population by 54–80%, thereby optimising resource allocation.^55^ Emerging biomarker-based algorithms have been used in Thai populations: a Markov microsimulation using Thai data demonstrated that GAAD-based surveillance was superior to ultrasound plus AFP, delivering better outcomes at lower cost (ICER: US$4631/QALY), within the national willingness-to-pay threshold.^56^

Egypt’s Ministry of Health launched a formal national HCC surveillance and management program in 2019, providing free lifelong monitoring for all patients with liver cirrhosis regardless of aetiology. Implemented through a network of 58 specialized units across the country. Cirrhosis patients (identified by FIB-4 ≥ 3.25, liver stiffness >12.5 kPa, or biopsy) are enrolled and followed every four months with ultrasound and AFP.^57^

Several countries have adopted more frequent surveillance intervals; whilst studies have shown benefit of 6-monthly ultrasound versus longer intervals, there is no evidence supporting the use of further shortened intervals.^58^ It would be of interest to retrospectively observe the impact of the shortened surveillance interval on rates of diagnosis and compliance. Moreover, whilst an area of ongoing research, there is currently no prospective, randomised research supporting the use of alternate imaging strategies in super-high-risk patient groups (as defined by Japan).

### USA

The AASLD recommends surveillance in patients with cirrhosis and high-risk subgroups with chronic HBV infection (Table 2).^7^ However, some centers have extended the surveillance population to include MASLD and advanced fibrosis, use alternate imaging modalities such as MRI and multi-phase CT and use a variety of biomarkers, reflective of a lack of national programme.^59^

Underuse of surveillance is common, with less than 20% of at-risk patients in receipt.^60^ Notably, a dual-center study found that over 50% of patients without surveillance in the year prior to HCC diagnosis were attributed to lack of any regular outpatient care.^61^ Surveillance utilization is lower among patients followed by primary care providers alone, which is common in rural areas and safety-net health systems given inadequate access to hepatology subspecialty care. Surveillance testing is covered by most insurance plans, although there can be out-of-pocket expenses related to co-pays, deductibles, parking fees, and opportunity costs for missed work.^29^ Approximately 10% of Americans are uninsured and others are underinsured. Registry data shows that those identifying as Black and from lower socioeconomic background are less likely to receive surveillance.^62^

### The patient experience

For people living with cirrhosis or other CLD, surveillance should offer reassurance and support early detection; yet it is often experienced as fragmented, uncertain, and anxiety-provoking. Evidence from a British Liver Trust survey of 135 people affected by primary liver cancer indicates that surveillance is frequently undermined by structural, informational, and emotional barriers.^63^

Communication gaps persist. Many patients do not understand the purpose, benefits, or limitations of monitoring, reflected in comments such as “my doctor gave me no advice whatsoever” and “I don’t really understand what the scan is for”.^64^ Without clear communication, surveillance may feel optional and difficult to prioritise.

Inconsistency in delivery further compounds these challenges. Although regular imaging is recommended, it is not reliably implemented; one carer reported that “the regular 6 monthly scans … never happened … too far advanced for any treatment”. Others describe being “forgotten by [the] liver team”. Delays in receiving results and poor coordination often add to anxiety. This can leave patients and carers to manage complex pathways themselves, increasing burden on those who often face complex circumstances and exacerbating inequalities.

Stigma remains an important barrier in liver disease. Many patients report feeling blamed or judged, with assumptions about alcohol use shaping engagement with care and contributing to delayed diagnosis of HCC.^65^

Access barriers persist: some reported waits exceeding six months and travel burdens of over 20 miles for hepatology care including surveillance.^63^ Surveillance must be reframed as a patient-centred pathway: consistent, clearly communicated, stigma-aware, and locally accessible and at times that suit the patient. Without this, it risks reinforcing inequalities rather than improving outcomes.

## Discussion

### Future direction: strengthening evidence, political willpower and targeted funding

A 2024 Delphi panel on HCC surveillance highlighted current challenges and future considerations to ensure that surveillance is optimised.^66^ There was consensus that the Chinese trial data was not generalisable to contemporary Western cohorts. All experts agreed that trials comparing different modalities of surveillance would offer useful information on the utility of surveillance on early-stage detection and mortality.

Europe is home to the highest concentration of high-income nations, well-established infrastructure and almost universal healthcare access free at the point of use for patients.^11^ Despite this, surveillance uptake and consequent late-stage HCC diagnoses mirror those of much resource-poorer nations. The 2022 *EASL Policy Statement on Liver Cancer Screening* highlights a key political and funding challenge: while professional bodies support HCC surveillance, both the European Commission’s Group of Chief Scientific Advisors and the United States’ National Cancer Institute conclude that there is insufficient evidence to support surveillance.^67^ This results in a lack of political and financial support for HCC surveillance, with no European nations having a dedicated national surveillance programme despite rising mortality and significant impact on health systems. Surveillance is now in deadlock: further observational studies will not convince sceptics, and another randomised controlled trial is widely considered unfeasible and unethical. However, as illustrated by Denmark and Japan, perhaps a more pragmatic approach is one of risk-based surveillance whereby those at highest risk are offered surveillance.

**Panel 2**

Blood based markers are currently being tested in phase III trials, including GALAD (Gender, Age, AFP-L3, AFP, and DCP), GAAD (Gender, Age, AFP, and DCP) and ASAP (Age, Sex, AFP, and PIVKA-II). The GALAD score has passed phase III validation illustrating increased cancer detection compared to AFP.^68^ In a multicentre validation study, the performance of GALAD was retrospectively tested on patients with viral and non-viral cirrhosis who went on to develop HCC. GALAD outperformed AFP in detecting HCC 12 months prior to diagnosis. Methylated DNA, is gaining traction with two methylated DNA panels (mt-HBT and HelioLiver) showing performance similar to GALAD in detecting early-stage disease.^69^^,^^70^ In a recent prospective multicentre validation trial, HelioLiver demonstrated superior sensitivity and non-inferior specificity compared to ultrasound in HCCdetection.^71^

Biomarkers have the potential to increase utilisation, especially if they offer convenience with ease of scheduling and point of care testing. There is high patient acceptability, with patients preferring biomarkers to ultrasound alone or AFP.^72^ However, the widespread adoption of biomarkers as part of national or global surveillance strategies is likely to be limited by cost-effectiveness. A high-performing test is insufficient as a surveillance tool if its cost acts as a barrier.

Abbreviated MRI (aMRI) is emerging as a promising alternative imaging modality and is being assessed in multiple prospective trials.^73^ Potential benefits include increased sensitivity, reduced cost and reduced time to complete when compared to full-sequence MRI.^73^ Questions remain as to which populations to include in aMRI surveillance: whether individuals at highest risk of developing HCC (viral hepatitis), or patients most likely to have suboptimal views on ultrasound (MASLD).^74^

The concept of risk stratification will become increasingly important in the context of increasingly scarce resources and providing precision medicine to a heterogenous patient cohort. There are several reported tools for the stratification of risk: serological models and imaging-based assessments. The aMAP score (measuring Age, Male, Albumin-Bilirubin and Platelets) has been validated in international cohorts with excellent discrimination for predicting risk irrespective of aetiology or ethnicity.^75^ However, its performance in prospective cohorts remains to be investigated. The FIB-4 index, widely used in estimating the severity of liver fibrosis, has moderate discriminant ability in predicting HCC occurrence in HBV.^76^ Other scores which have shown to be effective include the Toronto HCC risk index^77^ and image-based stiffness assessments such as FibroScan^78^ or Shear Wave Elastography.^79^ However, the optimal risk stratification test remains to be determined in prospective studies.

Globally, several solutions have been implemented (Table 3) to widen participation in surveillance programmes with most nations offering surveillance free at the point of care, identifying patients using existing viral hepatitis networks. However, there is a distinct paucity of formal trials to test the efficacy of interventions, with only the USA investigating the roles of via outreach, recall and patient navigation.

**Table 3:** Highlights solutions which have been used in different countries, attempting to address challenges relating to HCC surveillance.

Solution	Countries
National surveillance programme, usually building on existing viral hepatitis networks	Egypt, Japan, Taiwan
Surveillance free of cost to the patient	Egypt, Taiwan, Australia, most European countries
Annual education programme for clinicians	Japan
Annual education programme for patients	Japan
Risk stratification to reduce unnecessary surveillance	Denmark, Thailand
Access to free direct-acting antivirals	Egypt
Specific MASLD strategy	Taiwan
Nurse-based recall systems, mailed outreach	USA, Australia
Patient navigation	UK, USA, Australia

Low- and middle-income countries including Egypt, Pakistan and Thailand have taken a more pragmatic approach and demonstrated innovation in tackling public health crises related to liver disease. They report marked successes in the elimination of viral hepatitis (via cost-effective locally produced direct-acting antivirals) and now MASLD.^80^, ^81^, ^82^ In resource scarce publicly funded healthcare systems, much can be done to prevent the onset of liver disease. This includes universal hepatitis B vaccination of neonates which remains variable across Europe^11^ and the introduction of minimum unit alcohol pricing and sugar tax, all of which have been shown to have public health benefits beyond liver disease.^83^^,^^84^ Central to the current state of play is the role of the patient voice as presented by charity groups who play a key role in lobbying parliaments to implement change. In terms of research, funding and implementation, Europe has been left behind, and HCC outcomes reflect this. It is imperative that a cohesive voice between clinicians, academia and the public is formed to combat government inertia.

## Conclusion

HCC continues to have dismal outcomes in part due to late-stage presentation. Surveillance results in early diagnosis when disease is amenable to curative treatment, but barriers exist in both access and delivery. There have been successes globally, with many nations outside of Europe focusing on reducing the rates of viral hepatitis, the leading cause of HCC. Apart from a handful of countries (Japan, Taiwan, and Egypt), national surveillance programmes do not exist. The lack of national surveillance programme, scepticism around guidelines and uncertainty around chronic liver disease diagnosis all contribute to suboptimal surveillance uptake and a high proportion of patients being diagnosed with late-stage disease.

**Key recommendations**

Strengthening the evidence base for surveillance through a large, multinational risk-based clinical trial.

Further research into alternative surveillance modalities (novel biomarkers, imaging), with subsequent updating of guidelines to ensure surveillance tests are of high sensitivity, cost-effective and appropriate for contemporary cohorts.

Healthcare systems and governments should target barriers in access to care. This can be via:

• Cohesive surveillance strategy across all levels of healthcare
• Free surveillance at the point of care to alleviate financial barriers
• Patient navigation to target logistical and motivational barriers

Enhanced advocacy at the professional level: professional societies such as EASL and AASLD should collaborate with patient groups and charities to have open dialogue with the European Commission and National Cancer Institute to understand the evidence required to establish funding and political support for surveillance.

## Contributors

Conceptualisation: RS. Data curation: all authors. Methodology: RS, MQ. Supervision: RS. Writing—original draft: all authors. Writing—review and editing: PJ, MQ, RS.

## Declaration of interests

MB is on the advisory board for Abbvie, Mirium, Aligos, GSK, Gilead and Roche. AGS has served as a consultant or on advisory boards for Genentech/Roche, AstraZeneca, Eisai, Exelixis, Bayer, Merck, Elevar, Boston Scientific, Sirtex, FujiFilm Medical Sciences, Exact Sciences, Helio Genomics, Roche, Glycotest, ImCare, Curve Biosciences, DELFI, Mursla, and Universal Dx.
